# A Rare Case of Adult Cervicothoracic Cystic Lymphangioma Presenting as Neurogenic Thoracic Outlet Syndrome

**DOI:** 10.7759/cureus.56146

**Published:** 2024-03-14

**Authors:** Majed A Almourgi

**Affiliations:** 1 Surgery, College of Medicine, Taif University, Taif, SAU

**Keywords:** complete excision, thoracic outlet, neurogenic, cervicothoracic, cystic lymphangioma

## Abstract

Cystic lymphangioma (CL) is an uncommon congenital malformation of the lymphatic system, often occurring in the head, neck, or mediastinum, potentially causing compression symptoms like dysphagia or dyspnea, and in rare cases, neurogenic thoracic outlet syndrome (nTOS). This report details a case of a 38-year-old male with a four-year history of a left lower neck mass, experiencing tingling in his left forearm over the last six months. The examination revealed a left supraclavicular cystic mass, with imaging suggesting CL compressing neurovascular structures. The patient underwent successful complete surgical excision through a left supraclavicular approach. Histopathology confirmed CL, with no recurrence observed over 19 months. The case highlights that cervicothoracic CL with adult presentation can cause pressure symptoms including nTOS. It also underscores the role of a multimodal diagnostic approach to differentiate it from other neck masses and that a supraclavicular approach can effectively remove the cyst, especially when the lower extension is not deep and there is no surrounding inflammation, thereby leading to relieving pressure and preventing recurrence.

## Introduction

Cystic lymphangioma (CL) is an uncommon congenital malformation of the developing lymphatic system [1-4]. The commonest site of CL is the head and neck region, even extending to the mediastinum where it can rarely lead to pressure manifestations specially in adults [3]. The commonest presentation is an asymptomatic cystic mass, mainly seen in children and rarely in adults with a few reported cases in the literature on the adult form [4-7]. Cervical CL may cause compression symptoms as dysphagia or dyspnea; to the best of our knowledge, there are only two reported cases in the English literature on adult CL causing neurogenic thoracic outlet syndrome (nTOS) [1,3,6,7]. Cervicothoracic CL with adult presentation must be differentiated from other cystic neck lesions, with those arising from branchial clefts, thyroglossal duct, and lymph nodes [1,8]. Initial diagnosis is usually done by color Doppler imaging sonography (CDIS); however, contrast-enhanced computed tomography (CECT) and/or magnetic resonance imaging (MRI) would be the choice if thoracic extension is suspected to assess the lymphangioma and to determine the appropriate surgical approach [6,7,9-11]. Sclerotherapy using OK-432 may be used in cases of pediatric cystic lymphoma; however, in adults, specially if there is a pressure manifestation, complete surgical excision would be the treatment of choice [5]. Definitive diagnosis is done by a histopathological examination of the resected mass [5-8,10,11]. Here, we report a case of an adult male who presented with a cystic lymphangioma causing nTOS, with a review of the literature.

## Case presentation

A 38-year-old male presented to the thoracic surgery clinic with a left lower neck mass of four-year duration. It was gradual in onset, slowly increasing in size, quite painless all over the course, and not associated with other symptoms for three and a half years. However, in the last six months he manifested a tingling sensation over the ulnar distribution of his left forearm with no other neurological or vascular manifestations. There was no history of trauma or comorbidities. The family history was unremarkable. He works as a soldier, is married, and does not smoke.

On examination, the patient was apparently a healthy male with an average body built and normal vital signs. An examination of the neck revealed a single left supraclavicular oblong mass with normal overlying skin; it did not pulsate or move with deglutition. The swelling was non-tender, non-compressible, fluctuant, and the lower pole could not be palpated. An examination of the left arm revealed no color change, no edema, and normal skin temperature; capillary refill was normal, and the radial pulse was palpable and was not affected by Adson's test. There were no signs of sensory, motor deficit, or muscle wasting in the left upper limb. There was no enlargement of cervical, axillary, or other groups of lymph nodes.

**Figure 1:**
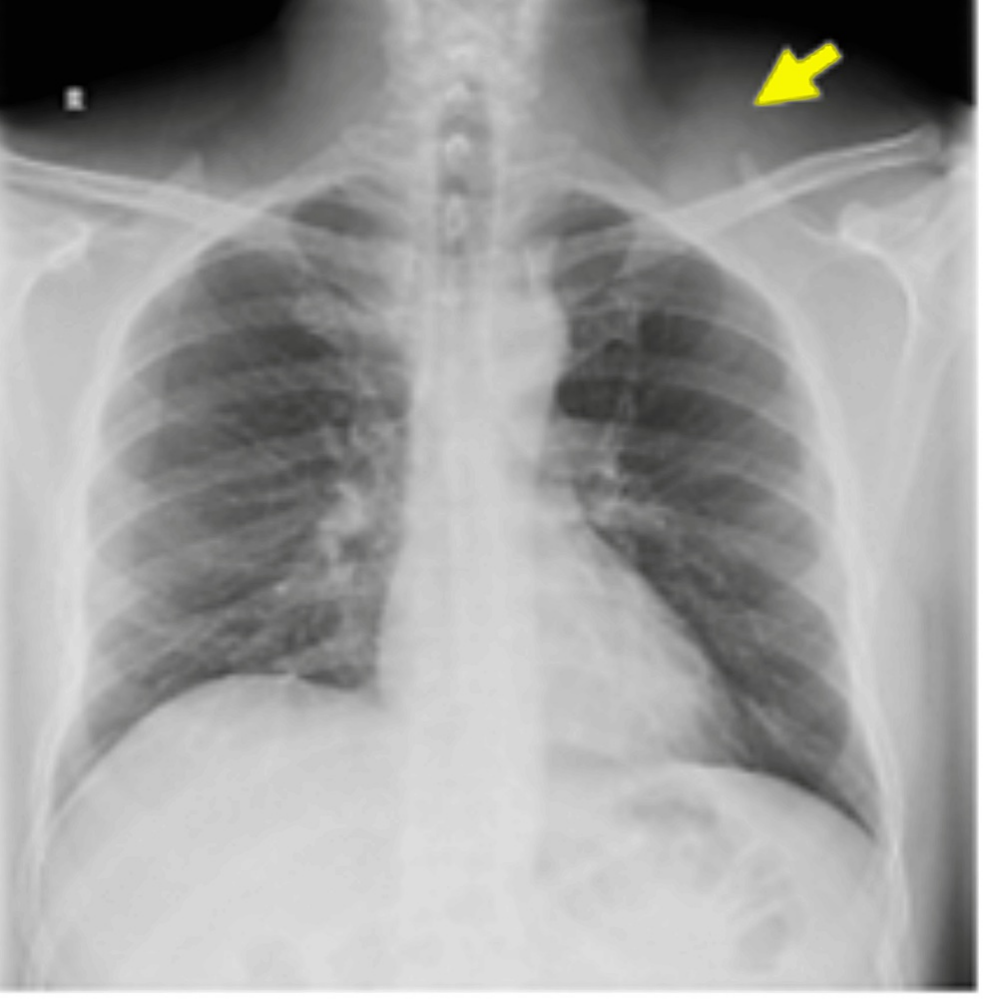
A plain radiograph of the neck and chest; the yellow arrow points to the soft tissue shadow in the left supraclavicular region.

**Figure 2:**
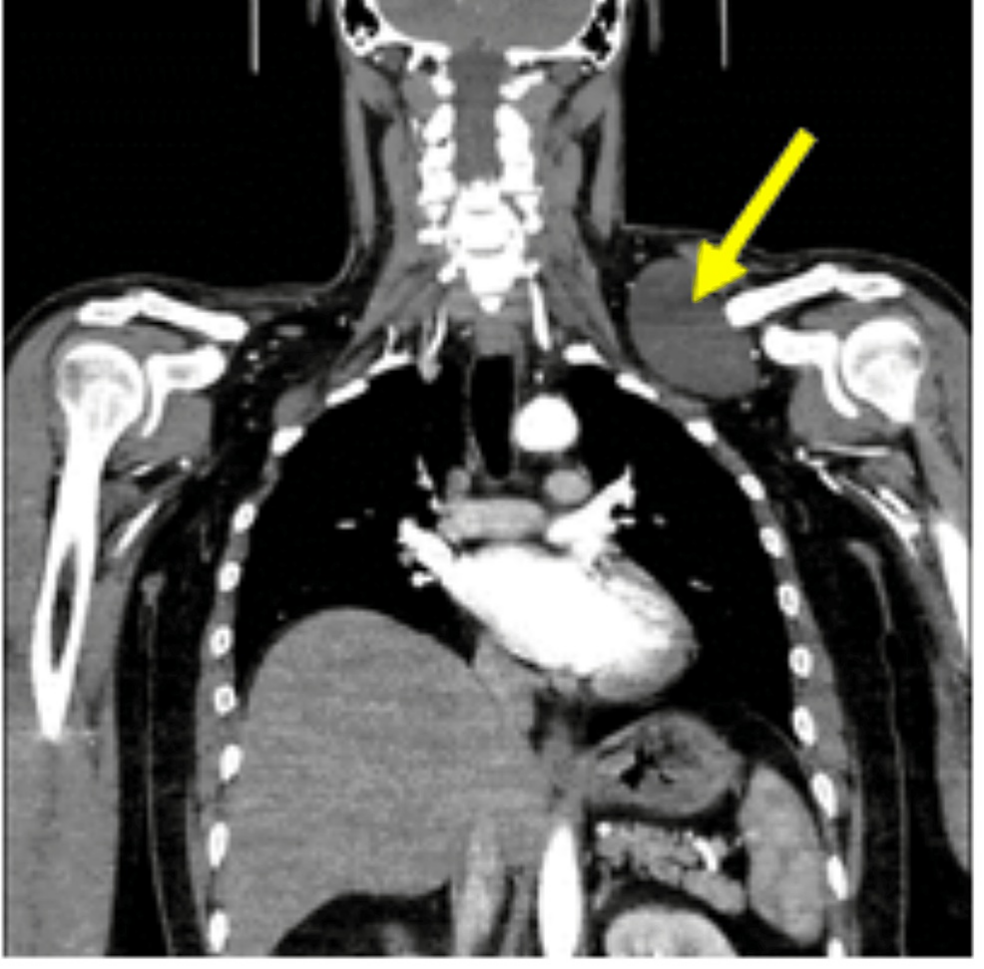
A CECT scan of the neck and chest; the yellow arrow points to the fluid containing the left supraclavicular lobulated mass extending to the apex of the left axilla. CECT, contrast-enhanced CT

**Figure 3:**
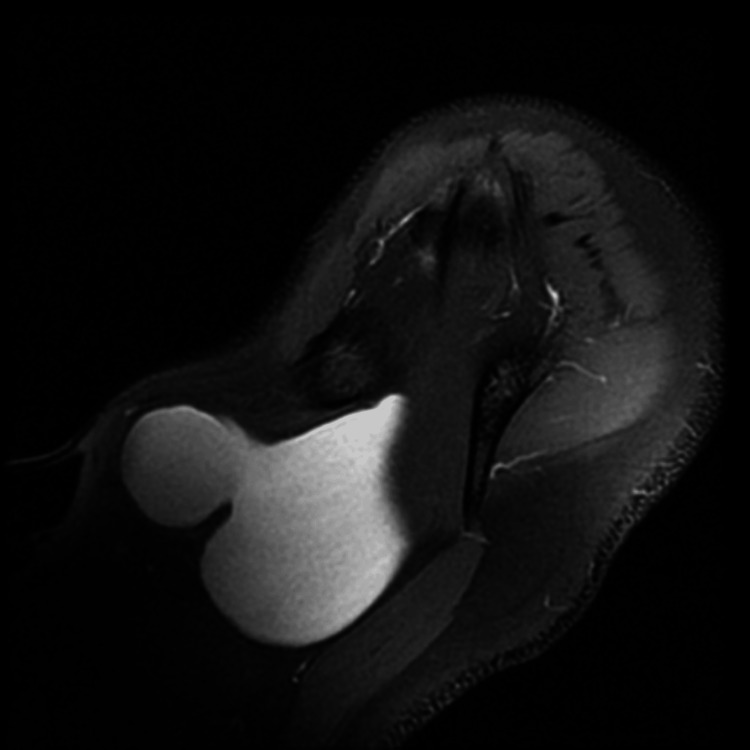
An MRI scan showing the lobulated cystic lesion seen in the left supraclavicular region deep to the trapezius muscle, insinuating between the clavicle anteriorly and the subscapularis muscle posteriorly.

Laboratory findings were normal. The neck radiograph showed a left supraclavicular soft tissue mass and no cervical rib (Figure 1). CDIS showed a finely trabeculated hypoechoic mass with patent nearby vascular axis. A CECT scan showed a left cervical mass extended to the infraclavicular space with a fluid component measuring 8 x 7.5 x 8.5 cm (AP x W x L), compressing the vascular structures alongside, in particular the jugular vein, which was pushed back and inwards; the laryngotracheal axis was intact, the left subclavian artery was not deviated, and there was no evidence of thrombosis (Figure 2). MRI showed a lobulated cystic lesion seen in the left supraclavicular region deep to the trapezius muscle, insinuating between the clavicle anteriorly and the subscapularis muscle posteriorly to end within the left axillary apex. It was T1W-hypointense and T2W-hyperintense with few fine septa, and no fat suppression or surrounding signs of oedema/inflammation (Figure 3). A cystic lymphangioma was suggested at this stage.

**Figure 4:**
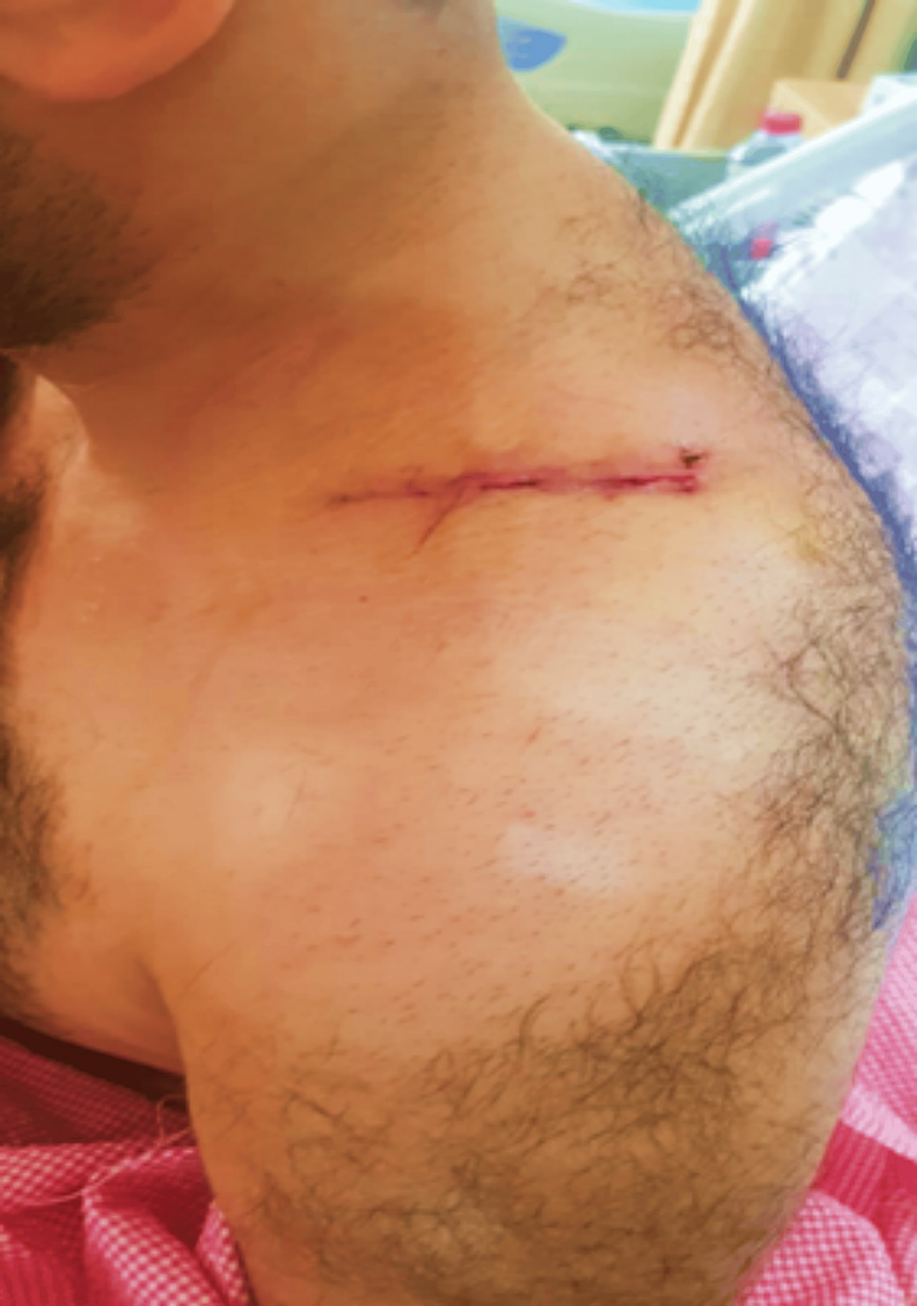
Postoperative photographs demonstrating the anteroposterior supraclavicular incision.

**Figure 5:**
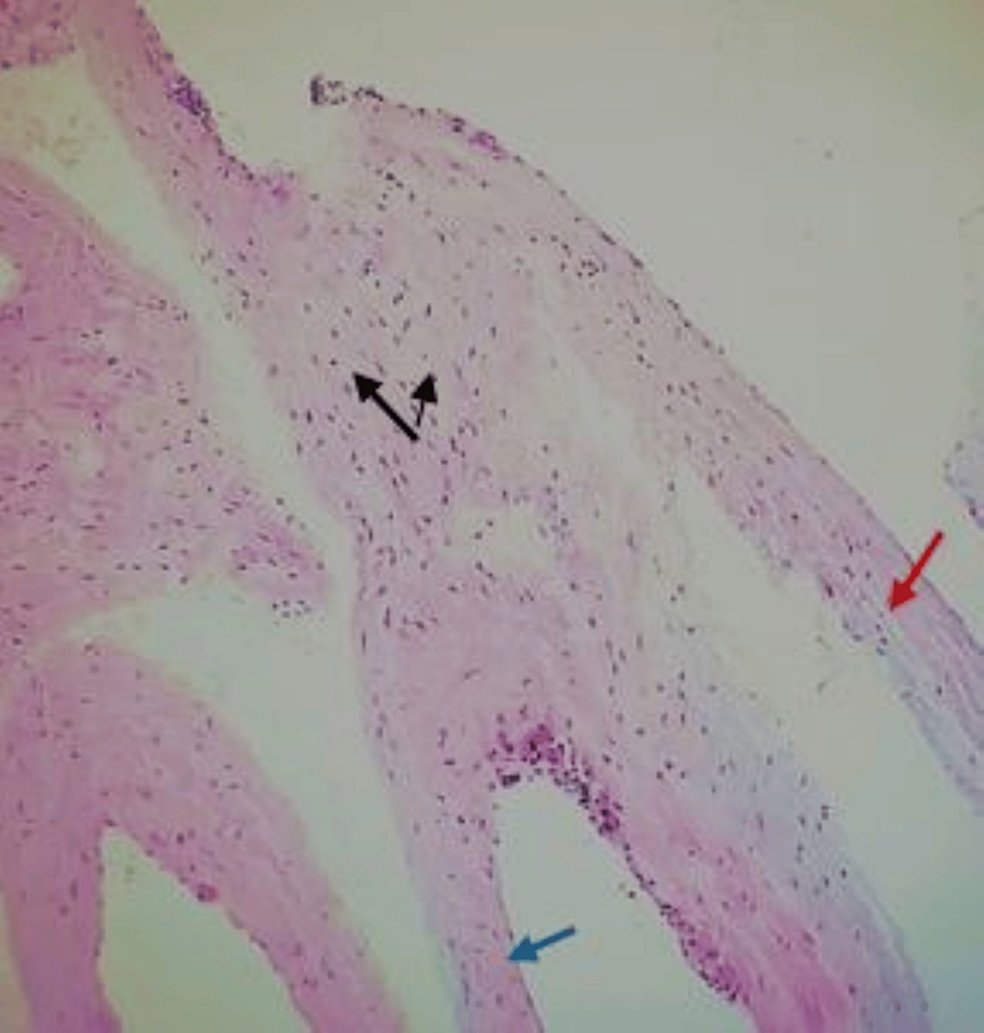
Several unequal thin-walled cavities (red arrow) are seen; the inner wall is lined with a monolayer of flat epithelial cells (blue arrow), the cavity is full of protein fluid, the endothelial cells are loose, the nucleus is small, and no atypia is found (black arrows) Hematoxylin and eosin stain, ×100

Complete surgical excision was the most appropriate treatment option, to relieve pressure and confirm diagnosis. The left anteroposterior supraclavicular approach was utilized (Figure 4). The cyst was exposed; it was tense and the absence of pericystic edema or inflammation allowed for easy mobilization and dissection of the cyst from the displaced muscles and neurovascular bundle. The lower pole was accessed readily, and complete excision was done without a need for a further exposing approach. The postoperative course was uneventful. The pathological report showed that the specimen had several unequal thin-walled cavities, the inner wall was lined with a monolayer of flat epithelial cells, and the cavity was full of protein fluid that confirmed the diagnosis of the cystic lymphangioma (Figure 5). The tingling sensation disappeared after surgery. At subsequent follow-ups over 19 months, no recurrence was found.

## Discussion

Cystic lymphangioma is an uncommon congenital malformation of the developing lymphatic system where the lymphatics fail to communicate with the central lymphatic vessels with or without deficient development of the venous system; it may be preceded by minimal trauma or respiratory tract infections [1-4]. CLs tend to surround and interlace between normal anatomic structures, even though they have no malignant potential [1]. CL can occur anywhere in the developing lymphatic system, especially in the head and neck region, even extending to the mediastinum where it can rarely lead to pressure manifestations especially in adults [3]. About 75% of cases are seen in the posterior triangle of the neck, and about 20% in the submandibular triangle, whereas the rest of cases can be detected in the mediastinum, axilla, and abdomen or even in multiple locations [5]. It presents commonly as an asymptomatic cystic mass in children and most cases (over 80%) are discovered before the age of two years; however, it is rarely encountered in adults with a few reported cases in the literature [4-7]. Cervical CL may cause compression symptoms such as dysphagia or dyspnea [1,3]. Kaira et al. presented a case series of adult cystic lymphangiomas where the commonest presentation was a cystic neck mass associated with mild pain and dysphagia; they also reported that one of the cases presented with a sudden increase in the size of a pre-existent swelling [5]. Marshall and Oliveira reported a case of an adult onset cervicothoracic cystic lymphangioma causing neurogenic thoracic outlet syndrome in a young collegiate athlete [6]. The second case reported in the English literature on adult CL with nTOS was by Vaidya and Vaithianathan [7]. To the best of our knowledge, the present case will be the third one describing CL with neurogenic TOS, in the English literature.

Adult cystic lymphangiomas must be differentiated from other cystic neck lesions, with those arising from branchial clefts, thyroglossal duct, and lymph nodes [1,8]. Initial diagnosis is usually done by CDIS where a trabeculated hypoechoic mass is seen. However, CECT and/or MRI would be the choice specially if thoracic extension is suspected, to assess the lymphangioma and to determine the appropriate surgical approach [6,7,9-11]. In accordance with the previous studies, the initial diagnosis of our case was done using US, followed by CECT and MRI; however, plain x-ray radiographs were done to exclude the presence of cervical rib that may cause nTOS. Kaira et al. advocated the use of fine-needle aspiration cytology (FNAC) to support the radiological diagnosis of a cystic lymphangioma and help to exclude malignancy [5]. This diagnostic tool was not used in our case and the other similar cases reported in the literature [6,7]. Sclerotherapy using OK-432 is recommended by many authors to be the initial treatment for pediatric cystic lymphomas; however, due to the rarity of adult CLs, there is no consensus about the use of sclerotherapy in adults [5]. In agreement with the findings of other authors, Gow et al. affirmed in their report that sclerotherapy would induce an intense immune response with a rapid increase in the size of the lesion and the risk of intolerable serious obstructive manifestations; hence, complete surgical excision is preferred in adults, particularly, if the lesion is related to major structures and/or is associated with pressure manifestation at the time of presentation [4-7,12,13]. Schefter et al. and Naidu and McCalla reported that adult CLs are commonly well circumscribed and complete surgical excision is easy and recurrence would be minimal; they added that the confirmation of diagnosis by a histopathological examination is one of the main advantages of complete surgical excision [14,15]. Kaira et al. emphasized on the discussion of the possible complications of surgery with the patient before consent is obtained, which include the length of the scar that would depend on the size of the mass, possibility of injury of important structures, venous bleeding, wound infection that is rare in neck surgery, and possibility of recurrence [5]. Our case presented with a swelling that was followed by pressure manifestations; imaging revealed a well-circumscribed cervicothoracic mass ending at the apex of the axilla with no signs of edema or surrounding inflammatory signs. Hence, complete surgical excision was performed through an anteroposterior supraclavicular incision; dissection was easy and the lower pole was accessed without the need of a combined approach. Marshall and Oliveira reported the need of an axillary incision with first rib resection once the dissection became deep; in the other case, Vaidya and Vaithianathan also reported that a combined trans-axillary approach was carried out to mobilize the cyst adequately [6,7]. In the present case, the diagnosis was confirmed histologically, and the symptoms resolved completely with no recorded recurrence or any other complication after 19 months of follow-up; similar results were reported by Marshall and Oliveira and Vaidya and Vaithianathan [6,7].

## Conclusions

This case underscores the importance of recognizing cystic lymphangiomas in adults, a pathology often overlooked due to its rarity. A painless fluctuant neck mass, especially coupled with neurogenic symptoms should prompt consideration of this diagnosis and guide further diagnostic workup to distinguish cystic lymphangioma from other neck masses. The utility of multiple imaging techniques, including radiography, color Doppler imaging sonography, contrast-enhanced CT, and MRI, is highlighted emphasizing the need for a thorough evaluation to assess the extent of the lesion and its impact on adjacent structures. This comprehensive approach is vital for planning the surgical intervention and anticipating potential complications. This case demonstrates that complete surgical excision can lead to favorable outcomes, including the resolution of neurogenic symptoms and no recurrence over an extended follow-up period. The necessity of histopathological examination post-surgery is highlighted. It not only confirms the diagnosis but also ensures the completeness of the excision.

In summary, this case report contributes valuable insights into the clinical suspicion, diagnostic strategies, and surgical management of cystic lymphangioma, particularly in adult patients. It underscores the need for a multidisciplinary approach combining careful clinical evaluation, detailed imaging studies, and precise surgical intervention for optimal outcomes.
